# 1216. ASP Initiated Blood Culture Service Is Highly Accepted, Reduced Antibiotic Durations Associated with Good Safety Outcomes

**DOI:** 10.1093/ofid/ofad500.1056

**Published:** 2023-11-27

**Authors:** Geraldine Tingting Foo, Hui Hiong Chen, Jia En Wu, Ka Lip Chew, Erna G Santoso, Jyoti Somani

**Affiliations:** National University Hospital, Singapore, Not Applicable, Singapore; NUH, Singapore, Not Applicable, Singapore; National University Hospital, Singapore, Singapore, Not Applicable, Singapore; National University Hospital, Singapore, Singapore, Not Applicable, Singapore; National University Hospital System (Singapore), Singapore, Not Applicable, Singapore; National University Hospital (Singapore), Singapore, Not Applicable, Singapore

## Abstract

**Background:**

NUH is a 1200 bed academic medical center with quaternary medical services ; the ASP program was initiated in 2014. A 2020 review found 18.5% of bacteremic patients were on inappropriate antibiotics (ABX) or wrong duration, and the overall majority of bacteremias were gram-negative (*E .coli and Klebsiella*). Since out ASP program has been promoting shorter durations in general, we hypothesized that a blood culture service where recommendations for de-escalation and appropriate duration were given early would help shorten ABX durations in patients with uncomplicated bacteremias. On March 15, 2021, we initiated an ASP led blood culture (BC) service (SVC) which reviewed case notes of patients Monday to Friday as soon as blood culture (BC) susceptibilities were available. We report on the types of interventions (INV), acceptance rate, and the impact and outcomes of our BC SVX between patients in whom the INV was accepted versus rejected.

**Methods:**

Prospective data on BC SVC interventions, acceptance rate, and patient status at five days were collected. Antibiotic duration (IV and oral), Length of Stay (LOS) and 30-day mortality as well as Charlson Comorbidity Index (CCI) were determined from electronic medical records for all ASP BCSVX interventions between March 15, 2021- December 31, 2022. Statistical analysis was performed in excel and STATA.

**Results:**

790 INVs were made on 625 patients and 693 courses of ABX with an 83.9 % overall acceptance rate. De-escalate ABX and treatment duration were the most common INVs (Table 1). 92.5% of patients had improved or no change in Day 5 status when ASP INV was accepted compared to 84.3% when rejected (p=0.003). Mean days of ABX per course was 5.9 versus 7.3 (p= 0.008) in the accepted versus rejected groups. CCI and LOS were similar, while 30-day mortality almost reached significance in the accepted INV group (Table 2).

**Figure:**
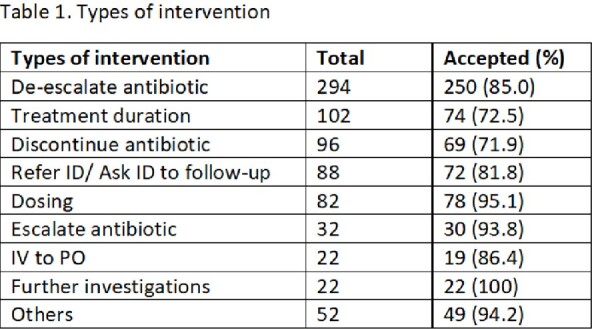

**Figure:**
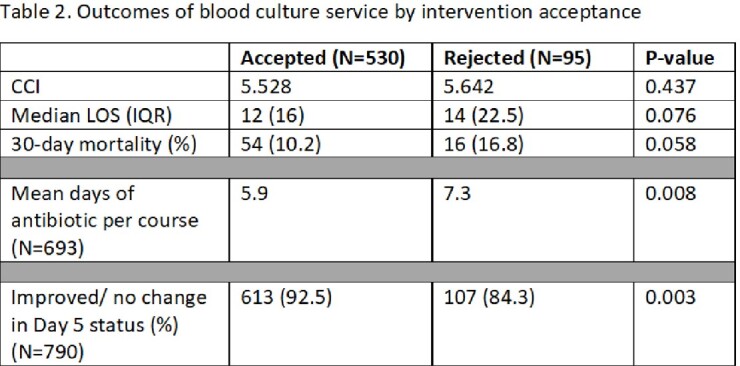

**Conclusion:**

ASP led BC SVC was well accepted, saved 2.14 days of ABX per intervention, and was very safe.

**Disclosures:**

**All Authors**: No reported disclosures

